# Influence of Trimethylamine *N*-Oxide on Platelet Activation

**DOI:** 10.3390/nu14163261

**Published:** 2022-08-10

**Authors:** Julian Josef Emonds, Clemens Ringel, Madlen Reinicke, Daniel Müller, Arnold Von Eckardstein, Jürgen Meixensberger, Uta Ceglarek, Alexander Gaudl

**Affiliations:** 1 Institute of Laboratory Medicine, Clinical Chemistry, and Molecular Diagnostics, University Hospital Leipzig, 04103 Leipzig, Germany; 2 Department of Neurosurgery, University Hospital Leipzig, 04103 Leipzig, Germany; 3 Institute of Clinical Chemistry, University Hospital Zurich, 8091 Zürich, Switzerland

**Keywords:** trimethylamine *N*-oxide, platelet activation, platelet lipidomics, thromboxane, HILIC-MS/MS

## Abstract

Microbiome-derived trimethylamine *N*-oxide (TMAO) has been associated with platelet hyperreactivity and subsequent atherogenesis. Whether physiological TMAO-levels influence platelet-derived lipid mediators remains unknown. Little is known about pre-analytic factors potentially influencing TMAO concentrations. We aimed at developing a quantitative LC-MS/MS method to investigate in-vivo and in-vitro pre-analytical factors in TMAO analysis to properly assess the proposed activating effect of TMAO on platelets. TMAO, betaine, carnitine, and choline were analyzed by HILIC-ESI-MS/MS within 6 min total run time. Method validation included investigation of reproducibility, recovery, sensitivity, and in-vitro pre-analytical factors. A 24-h monitoring experiment was performed, evaluating in-vivo pre-analytical factors like daytime or diet. Finally, the effects of different TMAO concentrations on platelet activation and corresponding alterations of platelet-derived eicosanoid release were analyzed. The method showed high reproducibility (CVs ≤ 5.3%), good recovery rates (96–98%), and negligible in-vitro pre-analytical effects. The influence of in-vivo pre-analytical factors on TMAO levels was not observable within the applied experimental conditions. We did not find any correlation between TMAO levels and platelet activation at physiological TMAO concentrations, whereas platelet-derived eicosanoids presented activation of the cyclooxygenase and lipoxygenase pathways. In contrast to previously published results, we did not find any indications regarding diet dependency or circadian rhythmicity of TMAO levels. Our results do not support the hypothesis that TMAO increases platelet responsiveness via the release of lipid-mediators.

## 1. Introduction

Microbiome-derived trimethylamine *N*-oxide (TMAO) rapidly acquired worldwide recognition for its potential associations with cardiovascular disease manifestations [1]. Recently, connections to numerous cardial and non-cardial pathologies have been reported [2]. TMAO is an amine oxide, found in animals and humans. Its precursors, quaternary ammonium compounds like betaine, choline, and carnitine, are found mainly in animal-based food sources, especially red meat, eggs, and dairy products [3]. Dietary precursors are metabolized by bacterial enzymes in the human intestine forming trimethylamine (TMA) [4,5,6], which is enterally absorbed. TMA is then oxidized to TMAO by hepatic flavin-containing monooxygenases (FMOs) and released into the bloodstream. Until its elimination via the kidney, the effects of TMAO in the human body remain ambiguous [7,8,9]. Associations of TMAO with atherosclerotic cardiovascular disease manifestations and potential pathomechanisms have been reported, like promotion of endothelial inflammation [10], increased oxidative stress [11], macrophage foam cell formation [1], and platelet hyperreactivity [12]. Great intra-individual variance [13], diet dependency [14], and indications of circadian rhythmicity [15] of TMAO plasma concentrations have been described. Hence, the utility of a diagnostic TMAO measurement from a single blood withdrawal has to be questioned and the analysis of possible in-vivo pre-analytical factors requires further research prior to assessing its functioning in physiological or pathological processes. Dietary effects on amino acid (AA) concentrations have been investigated [16] and can be used as a surrogate parameter for food intake in plasma.

The previously described TMAO-mediated increase in platelet responsiveness is thought to be caused by an augmented release of Calcium (Ca^2+^) from intracellular reservoirs, thereby promoting platelet aggregation and subsequent thrombus formation [12]. Whether these processes studied in vitro and in murine models have an effect in humans at physiological TMAO plasma concentrations remains to be clarified. For platelet activation monitoring, thromboxane B_2_ (TXB_2_) provides a proper surrogate marker. It is a stable degradation product of thromboxane A_2_ (TXA_2_), representing platelet activity in the TXA_2_-pathway [17,18] and a standard parameter for monitoring the effect of aspirin in patients [19]. TXA_2_ is metabolized from arachidonic acid via the cyclooxygenase (COX) [19,20]. In platelets, TXA_2_ enhances activation in a self-reinforcing cascade and thus provides for a thrombus formation [19]. COX is also responsible for the production of other metabolites from the groups of eicosanoids, like prostaglandins [20]. The evaluation of further platelet-derived lipid mediators in platelet aggregation is a subject of current research. Here, the response of other lipidemic enzymes, besides COX, such as lipoxygenases (LOX) or cytochrome P450 (CYP), to platelet activation is of great interest.

In the present study, we aimed at establishing a liquid chromatography-tandem mass spectrometry (LC-MS/MS) method for the simultaneous quantification of TMAO and its nutritional precursors betaine, carnitine, and choline in human plasma samples. In order to properly assess effect mechanisms, in vivo and in vitro preanalytical factors like diet, circadian rhythm, and storage conditions were to be analysed. In a final step the effects of TMAO-levels on platelet-derived lipid-mediators were to be investigated. To that end an experimental protocol for platelet activation in physiological and TMAO-spiked plasma samples was to be established followed by the monitoring of a subsequent release of an eicosanoid-panel (including TXB_2_) [21,22].

## 2. Materials and Methods

### 2.1. Quantification of TMAO and Its Precursors

#### 2.1.1. Chemicals and Reagents

LC-MS grade methanol, acetonitrile, and formic acid were purchased from Biosolve (Valkenswaard, The Netherlands). Water was provided by inhouse purification using a Barnstead Nanopure from Thermo Scientific (Waltham, MA, USA). Furthermore, LC-MS grade water was purchased from Biosolve (Valkenswaard, The Netherlands), VWR (Darmstadt, Germany), Roth (Karlsruhe, Germany), Sigma-Aldrich (St. Louis, MO, USA), Th.Geyer (Renningen, Germany), Fisher Scientific (Schwerte, Germany). Sodium chloride, ammonium formate, betaine, choline chloride, choline-d9 chloride, TMAO, TMAO-d9 were purchased from Sigma Aldrich (St. Louis, MO, USA). Carnitine was purchased from Sigma Aldrich (St. Louis, MO, USA). Deuterated standards betaine-d9, carnitine-d3 were purchased from CDN Isotopes (Pointe-Claire, QC, Canada). Aqueous working standards were produced for all analytes and internal standards based on 1 mg/mL stock solutions. A six point in house calibration was produced in NaCl solution covering 5.3–213 µmol/L for betaine, 2.8–112 µmol/L for carnitine, 2.4–96 µmol/L for choline, and 0.3–13 µmol/L for TMAO. Ammonium formate buffer solution (150 mmol/L, pH = 3.3) was prepared by diluting 9.46 g ammonium formate in 985 mL water and adding 15 µL formic acid. For the purpose of validation and quality control, a plasma pool was spiked to obtain samples at native and elevated concentrations (for final added concentration see Supplementary Table S1).

#### 2.1.2. Human Samples

Residual material of human blood was taken from patient’s routine diagnostics of the University Hospital Leipzig (ethical approval 082-10-190-42010). Samples were stored at −80 °C prior to analysis.

#### 2.1.3. LC-MS/MS

Aliquots of 10 µL of calibrators, quality controls, serum or plasma were treated with 90 µL precipitating agent (methanol including the internal standards), thoroughly mixed, and centrifuged for 5 min at 13,000× *g*. Ten µL of the supernatant were transferred to autosampler vials and further diluted with 990 µL of eluent B (ACN/150 mmol/L ammonium formate buffer 90/10 *v*/*v*) resulting in an overall dilution factor of 1000. A Prominence UFLC system from Shimadzu (Duisburg, Germany), consisting of two high-pressure gradient pumps (LC20ADXR), a column oven (CTO20AC), and the controlling module (CBM20A) was coupled to a QTRAP^®^ 6500 from SCIEX (Framingham, MA, USA). A PAL HTS-xt autosampler from CTC Analytics (Zwingen, Switzerland) handled sample injection. Chromatographic separation utilized a Kinetex^®^ HILIC column (2.6 µm, 50 × 2.1 mm) from Phenomenex (Darmstadt, Germany) at a flow rate of 1.0 mL/min yielding a total run time of 6 min. The mobile phase consisted of 100% eluent B (ACN/150 mmol/L ammonium formate buffer 90/10 *v*/*v*) and 0% eluent A (H2O/ACN/150 mmol/L ammonium formate buffer 80/10/10 *v*/*v*/*v*) and was adjusted as follows: 0–0.5 min 100% B, 0.5–3.1 min from 100% to 74% B, 3.1–3.2 min from 74% to 0% B, 3.2–4.0 min 0% B, 4.0–4.1 min from 0% to 100% B, 4.1–6.0 min 100% B. The column oven was tempered at 40 °C and the injection volume was 5 µL. Electrospray ionization (ESI) was applied in positive mode. Mass transitions for all analytes and internal standards as well as ion source parameters are given in Supplementary Table S2.

#### 2.1.4. Method Validation

LLOQ and LOD were estimated from at least five individual plasma samples as concentrations at signal/noise of 10 and 3, respectively. Linearity of the calibration was proven by regression analysis. Means of slopes and regression coefficients of ten six-point calibrations were determined for the robustness of linearity. Repeatability and intermediate precision, as well as recovery, were determined by measuring replicates of a plasma pool at native as well as two elevated concentration levels. Spiked concentrations for validation and control material are given in Supplementary Table S1. To assess trueness of the measurement, 30 EDTA plasma samples, as well as two quality controls and six calibrators, were sent to the Institute of Clinical Chemistry (IKC) of the University Hospital Zurich for method comparison [23]. To investigate preanalytical factors in vitro, whole blood drawn from three individuals were stored for 30, 60, 90, and 120 min at room temperature and at 4 °C, respectively, prior to centrifugation. The influence of sample material was investigated by comparing EDTA plasma, citrate plasma, lithium-heparin plasma, and serum. Freeze-thaw stability (−80 °C) was tested using three aliquots of pooled plasma.

### 2.2. TMAO In-Vivo Pre-Analytics

#### 2.2.1. Participants and Exclusion Criteria

An observational 24-h monitoring experiment was performed on healthy volunteers (n = 12) on two consecutive days at the Institute of Laboratory Medicine, Clinical Chemistry and Molecular Diagnostics (ILM) of the University Hospital Leipzig, Germany. We excluded participants with (1) age less than 18 years, (2) a history of chronic diseases of heart, kidney, or vessels of any kind, (3) acute infection, (4) intake of antibiotics/probiotics less than four weeks before the experiment start, (5) intake of acetylsalicylic acid less than two weeks before the experiment start, (6) intake of fish/seafood, yoghurt, grapefruit, broccoli, sprouts, cabbage, cauliflower, or kale on the day before experiment day. The experiment was approved by the institutional ethics board of the Medical Faculty of the University of Leipzig (ethical approval 131/19-ek). Written informed consent was obtained from all participants.

#### 2.2.2. Sampling and Experimental Procedure

**Figure 1:**
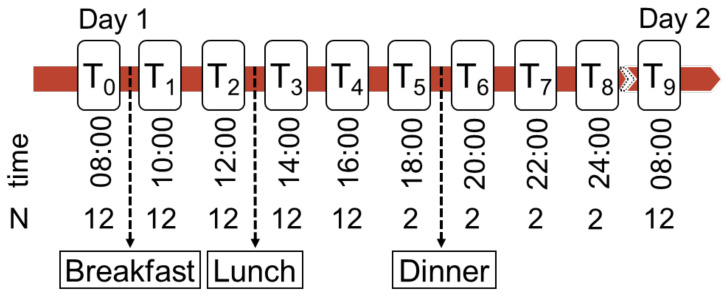
24-h monitoring experiment, timeline of blood sampling. Sampling times (T0–T9), number of participants (N) and meals during the sampling procedure over 24 h on two subsequent days.

EDTA-Plasma samples for the quantification of TMAO, betaine, carnitine, and choline were collected at nine different time points (T_0_–T_9_, Figure 1). For sampling points after overnight fasting (T0 and T9), after breakfast (T1 and T2), and after lunch (T3 and T4) blood from all 12 volunteers was taken, while at T5–T8 only two participants were monitored to obtain two extended profiles. All samples were centrifuged within 30 min and stored at −80 °C until measurement. Amino acid profiles were measured in plasma samples from all time points with a previously established method to monitor the dietary intake [16]. Additional fasting serum samples were collected at the start of the experiment (T_0_) for the measurement of routine laboratory parameters. Participants fasted for 12 h before the first (T_0_) and last sampling (T_9_). Breakfast was consumed between 8 am (T_0_) and 10 am (T_1_) and contained wheat roll, Gouda cheese, iceberg lettuce, cucumber, tomato, and banana for all participants. Lunch was taken between 12 am (T_2_) and 2 pm (T_3_) and contained pizza with vegetables. Dinner was taken independently by each participant between 6 pm (T_5_) and 8 pm (T_6_) and was not standardized but limited. No meal contained meat, fish, or any other ingredient listed as exclusion criteria. During the experiment participants only drank water, coffee, or tea. Information on medical history, medication and nutritional behaviour was gathered anamnestically.

### 2.3. Platelet Activation

#### 2.3.1. Participants and Exclusion Criteria

For platelet function analysis, platelet activation was performed on samples from different healthy volunteers at the ILM. We excluded all participants with (1) age less than 18 years, (2) a history of chronic diseases of any kind, (3) acute diseases of any kind, (4) intake of medication of any kind less than two weeks before the experiment starts. The experiments were approved by the institutional ethics board of the Medical Faculty of the University of Leipzig (ethical approval 236/18-ek). Written informed consent was obtained from all participants.

#### 2.3.2. Experimental Procedure

The experimental setup was based on a previously established and further developed protocol by Mütze et al. [21].

**Figure 2:**
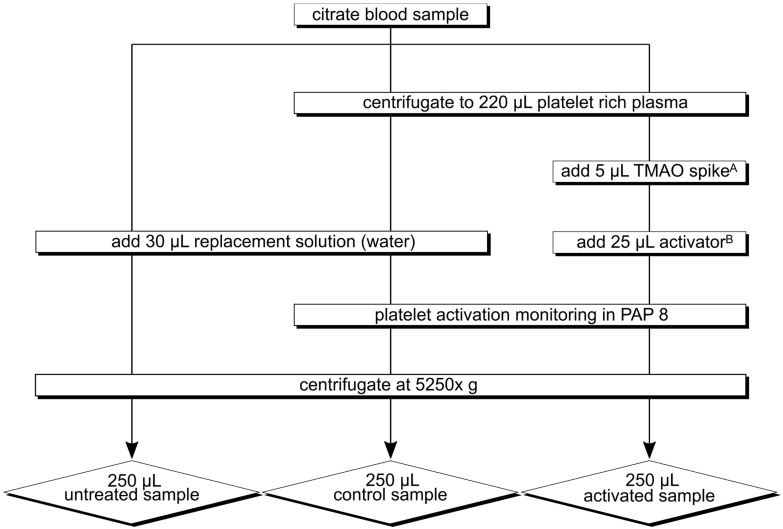
Platelet activation experiment according to Born, experimental setup and sample types. Citrate blood was sampled from healthy participants. TMAO indicates trimethylamine-N-oxide; PAP 8, platelet activation profiler 8. ^A^ TMAO solved in water (Target concentration in the sample: 0, 5, 20, or 100 µmol/L); used in experimental series 3 only. ^B^ ADP (5 µmol/L; used in experimental series 3 only), Ristocetin (1.2 mg/mL).

Blood samples were collected in tubes containing citrate. All samples were processed within 30 min after their collection. Samples were centrifuged to platelet-rich plasma (10 min, 200× *g*). Platelet activation was performed with ristocetin or adenosine diphosphate (ADP) on platelet-rich plasma and controlled by light transmission aggregometry according to Born using platelet aggregation profiler 8 (möLab, Germany) [24]. In experimental series 3 (Table 1), samples were spiked with TMAO (target concentration in the sample: 0, 5, 20, 100 μmol/L) prior to activation. Control samples went through all experimental steps, but no activators or TMAO spikes were added. Untreated samples were obtained at the beginning of the experiment from previously centrifuged platelet-poor plasma (15 min, 1500× *g*). Finally, all samples were centrifuged (10 min, 5250× *g*) and the supernatant was stored at −80 °C until further analysis (Figure 2). TMAO, betaine, carnitine, and choline were measured in all samples with the newly established LC-MS/MS method. For the determination of platelet activity, TXB_2_ was quantified by a well-established lipidomic LC-MS/MS assay for eicosanoids, which also delivered quantitative data on eicosanoids of LOX, COX, and CYP metabolism [22].

#### 2.3.3. Experimental Series

We performed three experimental series with samples of one participant and repeats on nine subsequent days (experimental series 1), seven participants and one repeat (experimental series 2), and one participant and three repeats in TMAO-spiked samples (experimental series 3, Table 1).

### 2.4. Statistical Analysis

Continuous variables are presented as medians (interquartile range) unless specified otherwise, whereas categorical and dichotomous variables are presented as numbers and percentages. Variables were tested for normal distribution using the Shapiro–Wilk test. Differences between sampling times or sample types were calculated using the Wilcoxon-Test for non-normally distributed samples, whereas differences between the groups were calculated using the Mann-Whitney U test for non-normally distributed continuous variables and the Chi-square test for dichotomous variables. *p*-values of less than 0.05 were considered statistically significant. Correlations between parameters were calculated using Spearman’s rank test for non-normally distributed data. Delta TXB_2_ was calculated by subtracting the concentration in the control samples from the concentration in the activated samples. Statistical calculations were performed using IBM SPSS statistic 20 (Armonk, NY, USA).

## 3. Results

### 3.1. LC-MS/MS

Minimal sample preparation via simple protein precipitation followed by dilution with eluent B was successfully applied. Having 15 mmol/L of ammonium formate buffer in the mobile phase was found to deliver efficient chromatographic separation and reproducible peak shapes within the short run time of 6 min. Unexpectedly high background noise was observed for betaine mass transitions. Water from in-house purification was identified as the source of said noise. Hence, LC-MS grade water from different producers was analyzed for their potential impairment. In all samples, betaine was found to be present in varying amounts (Supplementary Figure S2). Water producing the lowest amount of background noise was chosen as a constituent of the mobile phase. Initial validation, however, was affected by the described impairment. LLOQ (LOD) were calculated to be 0.78 µmol/L (0.23 µmol/L) for betaine, 0.10 µmol/L (0.03 µmol/L) for both carnitine and TMAO, 0.35 µmol/L (0.10 µmol/L) for choline. Linearity, as well as robustness of the inhouse calibration, was proven to show relative standard deviations of the slopes of the calibration curves below 6% (R^2^ ≥ 0.998). During validation CVs ≤ 7.8% were determined for all analytes with recoveries ranging from 90% to 111% for carnitine and TMAO while being slightly below 80% for betaine and choline. Following the resolution of the water issue, observation of quality controls showed improved precision (CVs ≤ 5.3%) as well as recoveries (96–98%) for all analytes (Table 2).

In short-term storage experiments of whole blood, choline concentrations increased by maximum 10% during 2 h at room temperature, whereas there was no increase observable at 4 °C. Betaine, carnitine, and TMAO did not show any concentration alteration at either storage condition (Supplementary Figure S3). All analytes showed high stability across five freeze-thaw cycles without exceeding the acceptable change limit (Supplementary Figure S4). The investigated material types showed similar analyte concentrations within the regular variance of the method except for choline in serum, which was slightly increased by 17% on average in relation to EDTA plasma (Supplementary Figure S5).

Basic results from the inter-lab comparison are given in Table 3 The null hypothesis of data similarity was valid by Mann-Whitney-U-Test. Means of the relative difference in quantitation in relation to IKC Zurich were +5.1% for betaine, −3.4% for choline, and −4.7% for TMAO with high correlation coefficients of R > 0.988 (Passing-Bablok regression and Bland-Altman plot can be found in Supplementary Figure S6). IKC Zurich did not analyse carnitine [23].

### 3.2. TMAO In-Vivo Pre-Analytics

#### 3.2.1. Baseline Characteristics

Baseline characteristics of the volunteers participating in the 24-h monitoring experiment are presented in Supplementary Table S3, including demographical characteristics, routine laboratory parameters, and nutritional behaviour. The median plasma concentration of TMAO at baseline was 2.7 (2.0–4.4) µmol/L and did not differ significantly between participants with a balanced or mainly vegetarian diet. Baseline TMAO did not correlate with routine blood parameters.

#### 3.2.2. TMAO, Precursors, and Amino Acids Progressions

Fasting concentrations of all measured amino acids did not present any interday changes between subsequent mornings. Plasma concentrations of most amino acids increased after food ingestion compared to their baseline fasting value, confirming the expected dietary effects. This postprandial increase in metabolite concentration could not be seen for TMAO (Supplementary Figure S8).

Changes in TMAO, betaine, carnitine, and choline plasma concentrations over 24 h are presented in Supplementary Table S4 and Supplementary Figure S7. TMAO and its precursors did not show any relevant change in concentrations between different sampling points, apart from a slight decrease of TMAO levels in the course of the morning. TMAO levels presented greater inter-individual variation between the 12 volunteers (median coefficient of variation over all sampling points: 37%) than intra-individual variation over time (median coefficient of variation of the 12 volunteers: 28%, Table 4).

#### 3.2.3. Correlation of TMAO, Precursors, and Amino Acids

Combined cluster correlation analyses of TMAO, its precursors, and amino acid concentrations showed no relevant correlations at baseline (T_0_) or any other sampling point—including post-prandial sampling points, while amino acids presented broad correlations among themselves.

Delta TMAO values of subsequent time points showed no significant correlation to delta amino acid concentrations. Only sporadic correlations between delta TMAO and changes in dietary precursor concentrations between sampling points T_0_ and T_2_ occurred.

### 3.3. Platelet Activation Experiment

#### 3.3.1. Thromboxane B2 and TMAO

**Figure 3:**
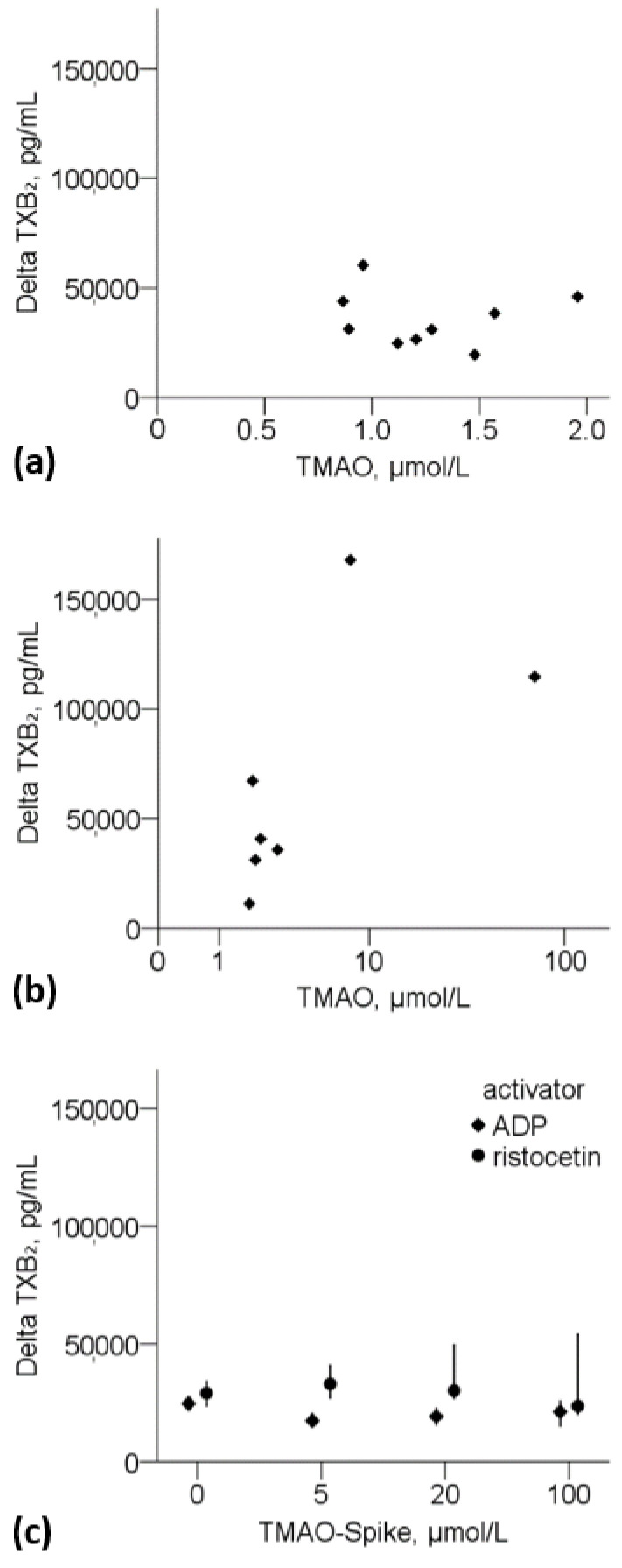
Platelet activation experiment, increase in TXB_2_ concentration (**a**) after platelet activation with ristocetin at physiological TMAO plasma concentrations (intra-individual variation; one subject, nine subsequent days; experimental series 1), (**b**) after platelet activation with ristocetin at physiological TMAO plasma concentrations (inter-individual variation; seven subjects; experimental series 2), (**c**) as median (95% CI) after platelet activation with ristocetin (dots) or ADP (squares) in TMAO-spiked samples (one subject, three experimental repeats; experimental series 3). TMAO indicates trimethylamine-N-oxide; TXB_2_, thromboxane B_2_.

In experimental series 1 (activation on nine consecutive days), TXB_2_ concentrations increased significantly (median TXB_2_-increase: 86-fold, IQR 58–103) after activation with ristocetin compared to control samples, while untreated and control samples did not differ in TXB_2_-concentrations (Supplementary Table S5). Baseline intra-individual variations of TMAO concentrations did not present an effect on the degree of platelet activation using TXB_2_-increase as an activation marker of COX-derived TXA_2_-pathway (Figure 3a). TMAO levels (untreated sample) and delta TXB_2_ did not correlate (Spearman’s ρ = −0.117, *p*= 0.765).

Next, we evaluated TXB_2_-increase after platelet activation in a cohort of seven participants (experimental series 2). Inter-individual baseline TMAO concentrations ranged from 1.8 to 68 µmol/L. TXB_2_ levels increased significantly (median TXB_2_-increase: 106-fold, IQR 46–185) after activation with ristocetin (Supplementary Table S6 and Figure 3b). There was no significant correlation between TMAO levels and delta TXB_2_ (Spearman’s ρ = −0.714, *p* = 0.071). Betaine, choline, carnitine and TMAO concentrations after activation differed not significantly compared to control samples or untreated samples of experimental series 1 and 2 (Supplementary Tables S5 and S6).

After activation, TXB_2_ in TMAO-spiked samples (experimental series 3) increased significantly for both ADP and ristocetin, with TXB_2_ concentration after activation with ristocetin being on average 44% higher than with ADP. We did not detect an increase in delta TXB_2_ concentration upon activation with ADP or ristocetin with increasing TMAO spike concentration (Figure 3c; Supplementary Figure S7).

#### 3.3.2. Eicosanoids

**Figure 4:**
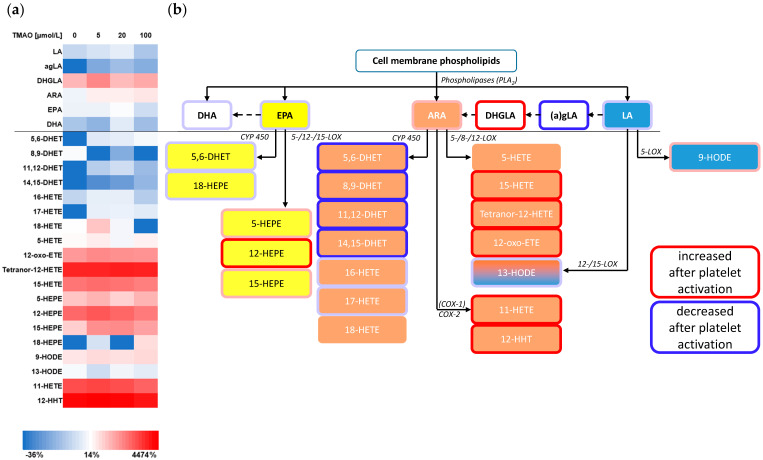
(**a**) Concentration-changes of eicosanoids after platelet activation with ristocetin at different TMAO-concentrations in relation to before activation. (**b**) Pathway classification of the quantified PUFA metabolites after platelet activation, including the most relevant enzyme classes. Concentration changes are marked using frames (red – increased, blue – decreased). The horizontal black line separates precursor fatty acids from metabolic products. TMAO indicates trimethylamine-N-oxide; a/gLA, α/γ-linolenic acid; ARA, arachidonic acid; COX, cyclooxygenase; DHA, docosahexaenoic acid; DHET, dihydroxy-eicosatrienoic acid; DHGLA, dihomo-γ-linolenic acid; EPA, eicosapentaenoic acid; ETE, eicosatetraenoic acid; HEPE, hydroxy-eicosapentaenoic acid; HETE, hydroxyl-eicosatetraenoic acid; HHT, hydroxy-heptadecatrienoic acid HODE, hydroxyl-octadecadienoic acid; LA, linoleic acid; LOX, lipoxygenase; PLA2, phospholipase A2.

To further investigate the effects of platelet activation and aggregation on platelet-derived lipid-mediators, besides thromboxanes, an extended eicosanoid panel was measured by LC-MS/MS in the samples before and after activation. Activation-related eicosanoid concentration changes showed similar patterns in all three series concentrations (Supplementary Tables S5–S7). Increasing concentrations of TMAO had no effect on the release of lipid mediators from ristocetin-activated platelets (Figure 4). Platelet activation with ADP showed comparable results.

In all platelet activation experimental series (1–3) a massive increase of COX-derived metabolites was detectable (e.g., 12-hydroxy-heptadecatrienoic acid (HHT), 11-hydroxy-eicosatetraenoic acid (11-HETE)). Relevantly increased concentrations were also shown for several LOX-derived metabolites like 5- and 15-HETE, 12-hydroxy-eicosapentaenoic acid (12-HEPE) or tetranor-12–HETE, while metabolites of the CYP-pathway (mainly the dihydroxy-eicosatrienoic acids (DHET)) showed decreased concentrations. For ARA and EPA, products of the LOX enzymes increased after platelet activation (e.g., 15-HETE), while CYP metabolites decreased (e.g., 11,12-DHET).

## 4. Discussion

TMAO is thought to increase platelet responsiveness via the release of intracellular Ca^2+^, which would support its proposed role in numerous associated diseases, particularly cardiovascular disease [12]. The increased incidence of thrombotic events has been demonstrated on several occasions [25,26], but associations of TMAO with the prevalence of atherosclerotic phenotypes could not always be demonstrated [27]. In order to further investigate said association, we established a HILIC-MS/MS method, assessed and standardized pre-analytical conditions, and performed platelet activation experiments.

### 4.1. LC-MS/MS

Due to high endogenous concentrations of the analytes, detection sensitivity was of no concern, and the focus of method development laid on robustness, selectivity, and analysis time optimization. With only 6 min total run time, the presented method passes by most of the current quantitative LC-MS/MS methods, maintaining high reproducibility as well as supposed high accuracy which was supported by the high degree of agreement in the inter-lab comparison [28,29,30]. The high resistance of the analytes against preanalytical influence renders sample drawing and storage straightforward in the context of clinical trials except for the slight increase of choline in short-term storage experiments of whole blood. This is in accordance with previous findings and has to be considered in the process of sample collection [31]. Encountering betaine contamination in in-house produced water as well as commercial LC-MS grade water led to the assumption of the whole process of purification being the culprit. In said process, deionizer resins among others are used to minimize the total organic carbon level. To that end, resins can be functionalized to contain quaternary amines and/or carboxylic groups. Betaine-bleeding ion exchange material would explain our findings and, to our knowledge, has not yet been described in the literature as an issue in the analysis of TMAO precursors. The improvement in precision and recovery using the least contaminated commercially available water underlines the importance of high purity method reagents.

### 4.2. In-Vivo Pre-Analytics

Broad intra-individual variations of TMAO plasma concentrations have been reported [13]. Hence, the pre-analytical question arises as to whether a TMAO measurement from a single blood withdrawal is potentially useful as a diagnostic biomarker. By analysing intra-day variability of TMAO plasma concentrations we could show, that TMAO plasma levels are not influenced by a circadian rhythm. We explain the detected decrease of TMAO plasma levels with a lack of intake of nutritional sources of TMAO that were excluded from the experimental diet and remaining circulating TMAO from food ingested prior to the observational period. Analysing postprandial amino acid profiles showed increasing levels after food ingestion, roughly confirming occurred digestion. TMAO however, did not increase postprandial and hence did not seem to be directly influenced by the nutritional intake during the experiment. Importantly, we found that over the course of 24 h, TMAO plasma concentrations presented greater inter-individual variation than intra-individual variation. Intra- and inter-individual variations of TMAO have been investigated over the course of a year [13] or on successive days [32], but progressions of TMAO-plasma levels over the course of a day and the corresponding proportion of intra- vs. inter-individual variations over this time-period have—to our knowledge—not been demonstrated before.

### 4.3. Platelet Activation

By means of robust LC-MS/MS analysis and negligible pre-analytical effects, in vivo as well as in vitro, the foundation for the investigation of the role of TMAO in platelet activity was built. Experimental series 1 and 2 show a significant increase in TXB_2_ concentration after activation with ristocetin. The magnitude of the increase is subject to slight inter-day variability in one individual (experimental series 1), whereas the inter-individual variability appears to be higher (experimental series 2). Since the change in TXB_2_ concentration (delta TXB_2_) does not show a significant correlation with TMAO concentration in experimental series 1, the variability of the TXB_2_ increase does not seem to be associated with TMAO. Single individuals from experimental series 2 with very high endogenous TMAO levels, however, showed a high release of TXB_2_ after activation compared to individuals with lower TMAO levels. Hence, a potential influence of TMAO concentrations on platelet activation was the subject of experimental series 3 in which there wasn’t any correlation observable between spiked TMAO levels and delta TXB_2_. This result suggests that TMAO does not increase platelet responsiveness in the short term. The possible relationship between TMAO and delta TXB_2_, that we observed in experimental series 2, could have arisen by chance due to the small sample size or disregarded influencing factors on TXB_2_ synthesis. A prolonged exposure time of TMAO in blood might also have an activating influence after all, which cannot be represented by the short-term addition of TMAO.

Thrombus formation is usually an amplifying cascade of signalling pathways, which we simulated using ristocetin or ADP [33]. In this process, arachidonic acid metabolites are converted by various enzymes, e.g., COX, LOX, and CYP, in the blood or blood cells. It has been described, that, in addition to thromboxanes, various eicosanoids are present in platelet metabolism and play a role in platelet activation, thrombus formation and progression of atherothrombosis [20,34]. We detected significant increases in the concentrations of products from the LOX and COX pathways, while products of CYP metabolism and early precursors predominantly decreased (Figure 4b). These effects appear to be enzyme-related but potentially independent from the precursor fatty acid. Their contradictory trends, however, do not allow for a conclusive interpretation. As a high-affinity ligand for BLT2 receptors, 12-HHT is produced in large quantities by activated platelets [35]. The increased production of 12-HHT and 12-HETE in human platelets suggests increased availability of free arachidonic acid, which was also previously reported through activation of GPR91 [36]. Induced changes to COX-products such as TXB2 and 12-HHT as well as LOX-derived metabolites after platelet activation have been described before, confirming our findings [37]. This further supports the thesis that triggering biological actions through platelet activation can reveal potential implications in thromboinflammation [38]. DHETs have been described as protective agents regarding endogenous mediation of vascular function in human coronary resistance vessels and vascular dilation [39], which might explain their decreased concentration in the presented experiments.

### 4.4. Limitations

During the 24-h-monitoring experiment food intake was standardized and the day prior to the experiment food sources high in TMAO were excluded from the diet. A questionnaire was used in order to determine food intake prior to the experiment. Nevertheless, the observed intra-individual variation of baseline TMAO levels may be due to food intake on the days before the experiment, which was not completely standardized. Another limitation of the study is the relatively small sample size in the experiments regarding pre-analytical factors and platelet-activation.

## 5. Conclusions

With the absence of a circadian rhythm, no direct effects of (vegetarian) diet, and lower intra- than inter-individual variation of TMAO plasma levels over the course of one day, we conclude that a blood withdrawal for the diagnostic quantification of TMAO can be done independently from time of day. Since we cannot exclude the possibility of effects of food sources not monitored in our experimental setup, a fasting state at the time of blood withdrawal seems advisable.

TMAO does not increase the responsiveness of platelets, concluded from the presented results under the given experimental conditions. Specific roles of individual metabolites of the discussed metabolic pathways remain to be clarified in contrast to the role of TMAO.

## Figures and Tables

**Table 1:** Platelet activation experiment, experimental series.

Experimental Series	Participants, n	Activator	Sampling
1	1	ristocetin	Samples were collected on nine subsequent days at the same daytime.
2	7	ristocetin	Samples were collected on different days at the same daytime.
3	1	ristocetin, ADP	Samples were collected on one day in three runs and spiked with different TMAO-concentrations

ADP indicates adenosine diphosphate; TMAO, trimethylamine *N*-oxide.

**Table 2:** Data on precision and recovery from validation experiments, as well as long-term quality control measurement. Shown data represents samples from validation (Val) as well as quality control (QC).

	Between Day				Within Run			
Betaine	Mean [µmol/L]	CV	Recovery	N	Mean [µmol/L]	CV	Recovery	N
Val native	29	5.6%		30	29	4.7%		30
Val spike level 1	63	6.2%	80%	30	66	4.4%	88%	30
Val spike level 2	96	6.8%	79%	30	97	5.6%	80%	27
QC native	28	4.6%		116				
QC spike level 1	111	3.8%	97%	96				
Carnitine	Mean [µmol/L]	CV	Recovery	N	Mean [µmol/L]	CV	Recovery	N
Val native	41	5.5%		30	40	4.1%		30
Val spike level 1	72	6.3%	101%	30	74	4.3%	110%	30
Val spike level 2	102	6.9%	99%	30	102	5.7%	100%	27
QC native	39	4.5%		116				
QC spike level 1	99	4.1%	96%	96				
Choline	Mean [µmol/L]	CV	Recovery	N	Mean [µmol/L]	CV	Recovery	N
Val native	9.2	7.8%		30	8.8	3.2%		30
Val spike level 1	17	6.2%	79%	29	17	3.8%	82%	30
Val spike level 2	31	5.8%	76%	29	30	4.7%	75%	27
QC native	8.6	4.9%		116				
QC spike level 1	56	3.9%	98%	96				
TMAO	Mean [µmol/L]	CV	Recovery	N	Mean [µmol/L]	CV	Recovery	N
Val native	4.0	7.8%		30	3.7	5.2%		30
Val spike level 1	5.2	6.3%	90%	30	5.2	5.0%	111%	30
Val spike level 2	10	6.9%	94%	30	10	6.5%	94%	27
QC native	3.7	5.3%		116				
QC spike level 1	10	4.8%	97%	96				

**Table 3:** Base data of inter-laboratory comparison given as median (IQR). Significance levels were calculated using Mann-Whitney U-Test.

	N	ILM Leipzig	IKC Zurich	*p*
Betaine [µmol/L]	31	29 (21–47)	26 (20–44)	0.678
Choline [µmol/L]	31	13 (7.3–29)	12 (7.7–28)	0.860
TMAO [µmol/L]	29	4.8 (3.0–10)	5.1 (3.3–10)	0.852

**Table 4:** Intra- and inter-individual variations of plasma concentrations of TMAO and dietary precursors.

Metabolite	Intra-Individual Coefficient of Variation ^1^	Inter-Individual Coefficient of Variation ^2^
TMAO	28%	37%
Betaine	7.3%	32%
Choline	11%	25%
Carnitine	6.5%	21%

^1^ Median coefficient of intra-individual variation over 6 sampling points within 24 h. ^2^ Median coefficient of inter-individual variation of 12 individuals within 24 h. TMAO indicates trimethylamine-*N*-oxide.

## Data Availability

The data presented in this study can be found in the Supplementary Materials or on request from the corresponding author.

## Supplementary Materials

The following are available online at https://www.mdpi.com/article/10.3390/nu14163261/s1, Figure S1: Chromatogram of a plasma sample showing all analytes and internal standards (IS), Figure S2: Measurement of water samples from seven different producers, Figure S3: Short term stability experiments, Figure S4: Freeze-thaw stability experiments, Figure S5: Matrix comparison experiment, Figure S6: Inter-laboratory comparison, Figure S7: 24-hour monitoring experiment, changes in metabolite concentrations, Figure S8: 24-hour monitoring experiment, changes in metabolite concentrations between T0 and T1, Table S1: Final added concentrations for samples used during validation (Val) as well as quality control (QC), Table S2: Mass transitions (m/z) of all analytes and internal standards, Table S3: 24-hour monitoring experiment, baseline characteristics, Table S4: 24-hour monitoring experiment, TMAO and its precursors at each sampling point, Table S5: Platelet activation experiment (experimental series 1), Table S6: Platelet activation experiment (experimental series 2), Table S7: Platelet activation experiment (experimental series 3).
