# Supplementary material for: Influence of Trimethylamine N-Oxide on Platelet Activation
Source: Nutrients. 2022 Aug 10;14(16):3261. doi: 10.3390/nu14163261 (PMC9413306; doi:10.3390/nu14163261)
Supplement: Supplementary file 1 [file nutrients-14-03261-s001.zip › nutrients-1799725-supplementary.pdf]

Supplementary Material

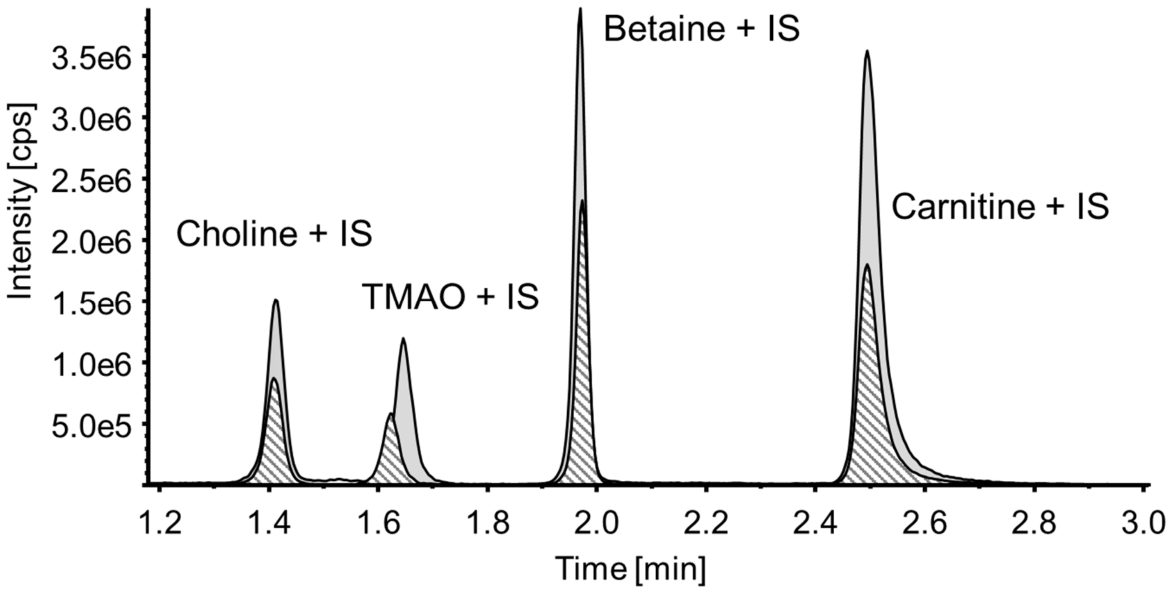

Figure S1. Chromatogram of a plasma sample showing all analytes and internal standards (IS). TMAO indicates trimethylamine *N*-oxide.

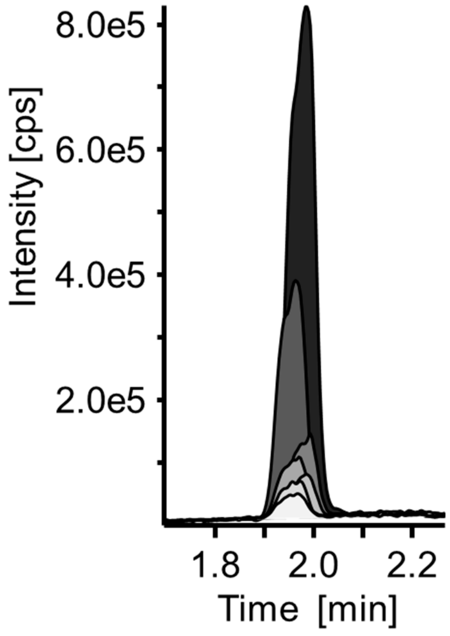

Figure S2. Measurement of water samples from seven different producers. Betaine mass transition is shown. Samples were injected directly without sample preparation leading to asymmetric peak shapes due to the high eluotropic strength of water in HILIC.

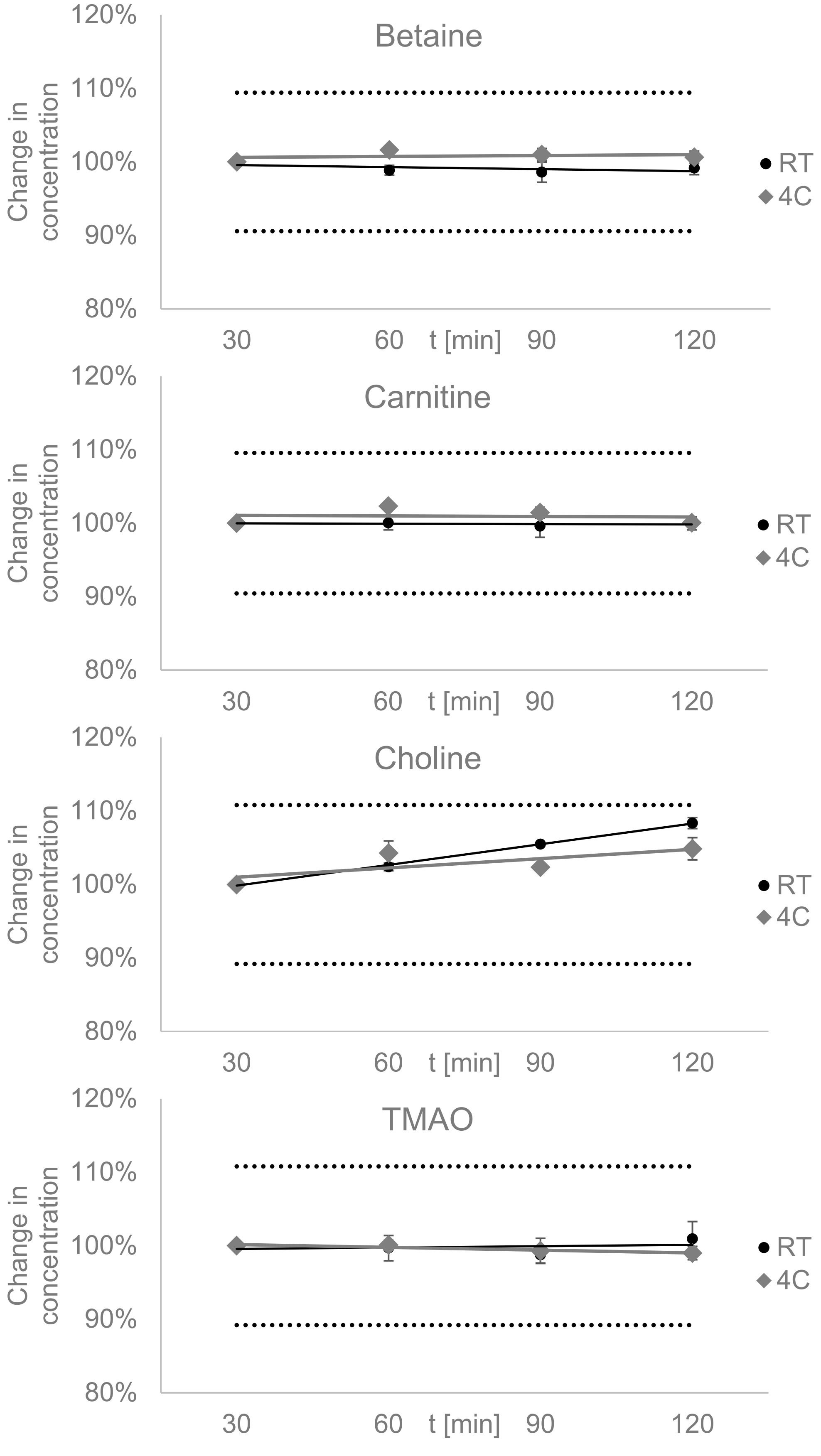

Figure S3. Short term stability experiments. Black circles represent room temperature. Grey diamonds represent 4°C. Solid lines represent linear fits. Dotted lines represent acceptable change limit (ACL = 2.77 times method coefficient of variation)

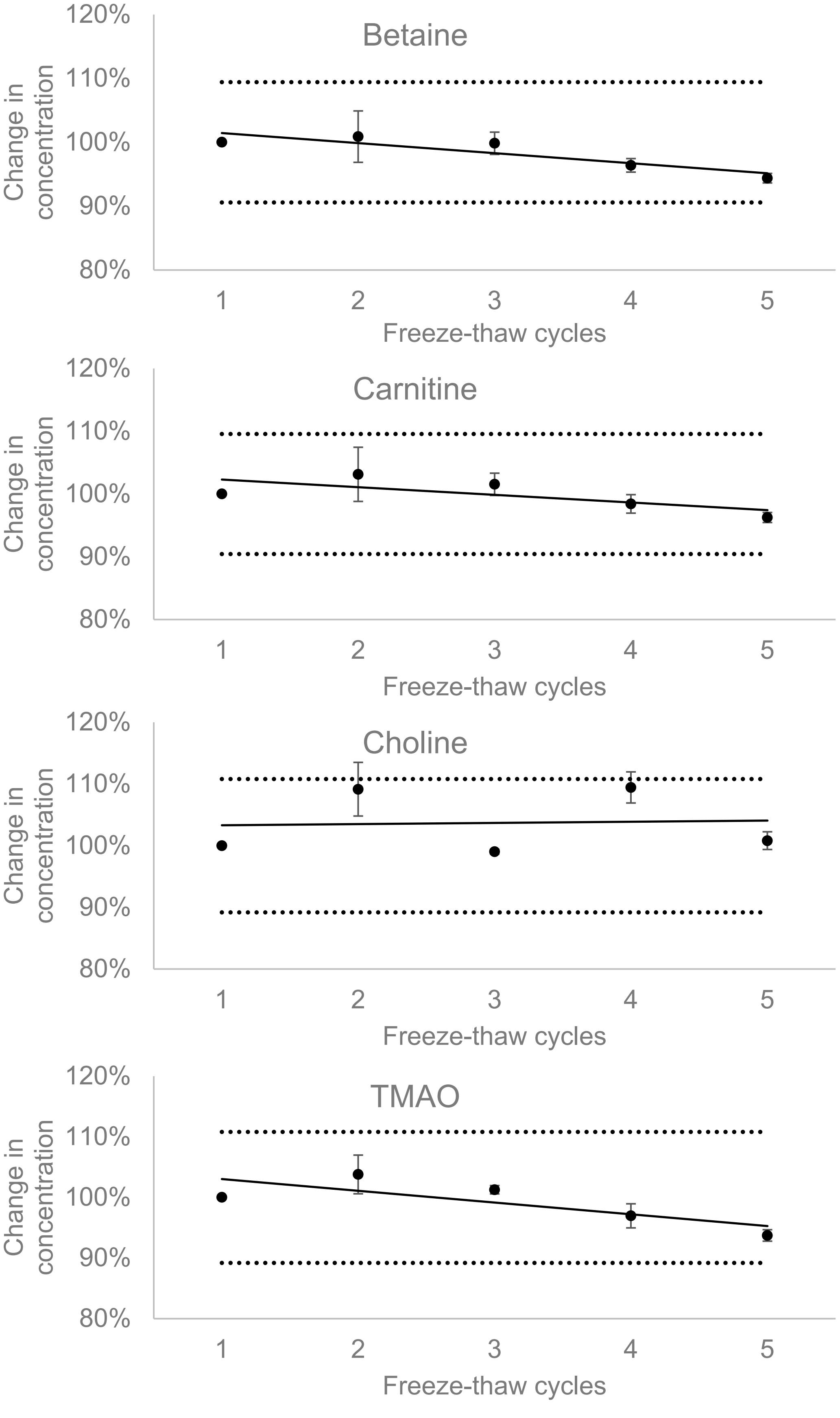

Figure S4. Freeze-thaw stability experiments. Black circles represent room temperature. Solid lines represent linear fits. Dotted lines represent acceptable change limit (ACL = 2.77 times method coefficient of variation)

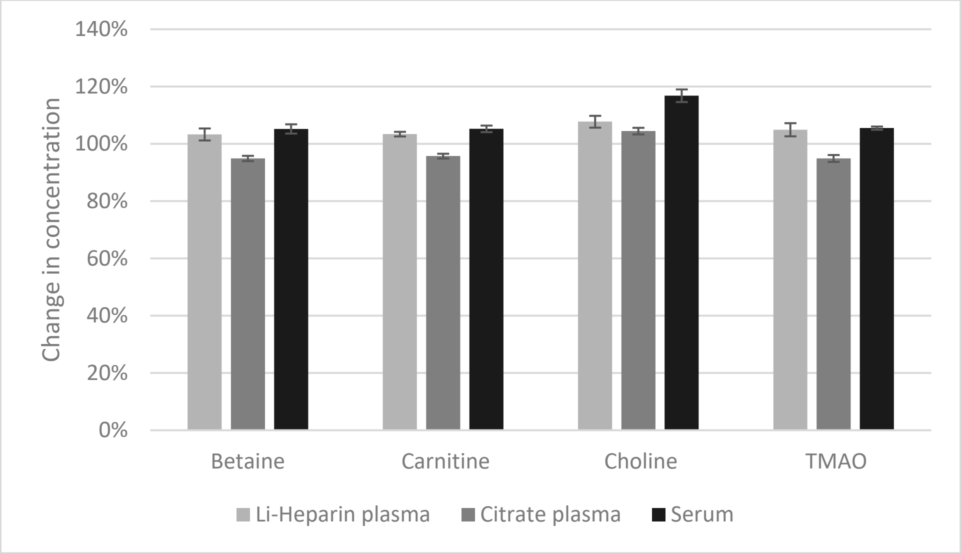

Figure S5. Matrix comparison experiment. Results are given in relation to EDTA plasma. 3 replicates from 3 individuals each were averaged to obtain the depicted results.

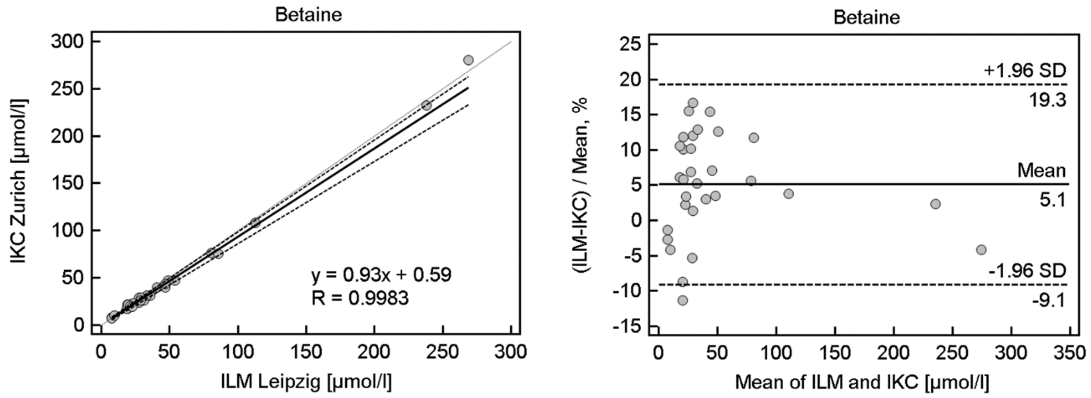

(a)

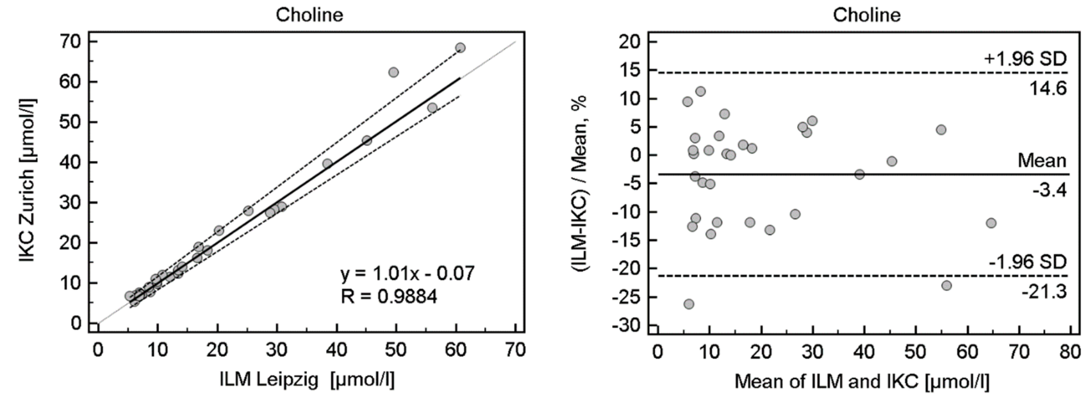

(b)

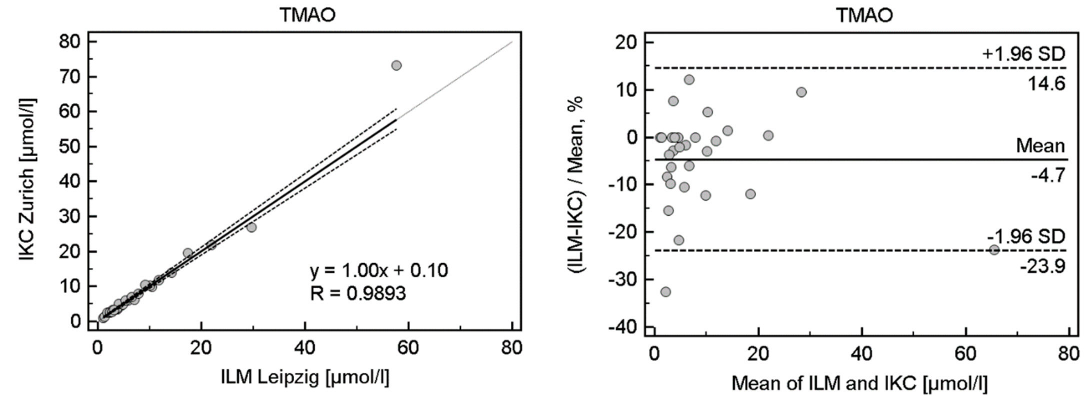

(c)

Figure S6. Inter-laboratory comparison (Passing-Bablok regressions and Bland-Altman plots) of LC-MS/MS methods for the quantification of **(a)** Betaine, **(b)** Choline, and **(c)** TMAO between Leipzig and Zurich.

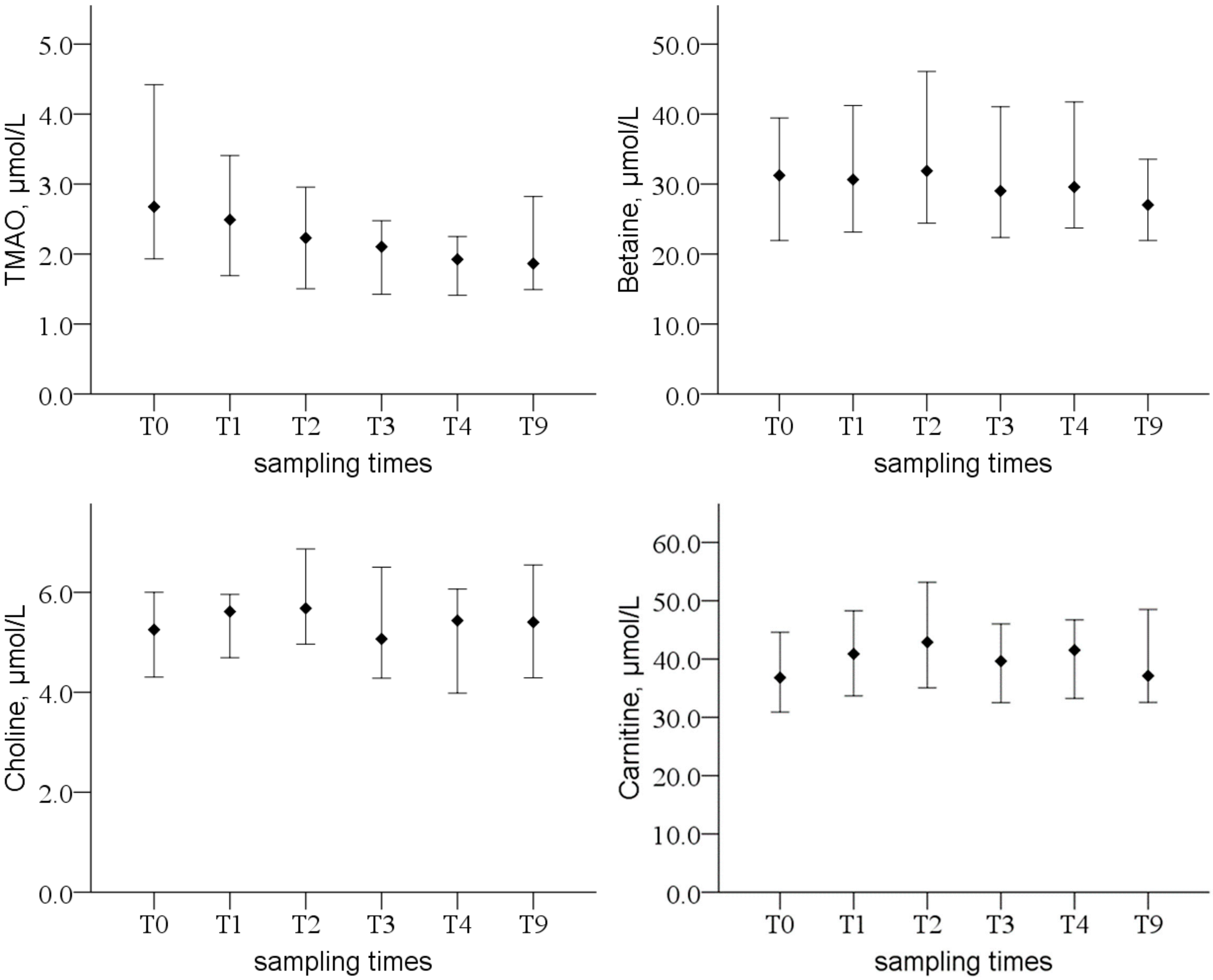

Figure S7. 24-hour monitoring experiment, changes in metabolite concentrations. Values are presented as median (95% CI) at each sampling time. TMAO indicates trimethylamine N-oxide.

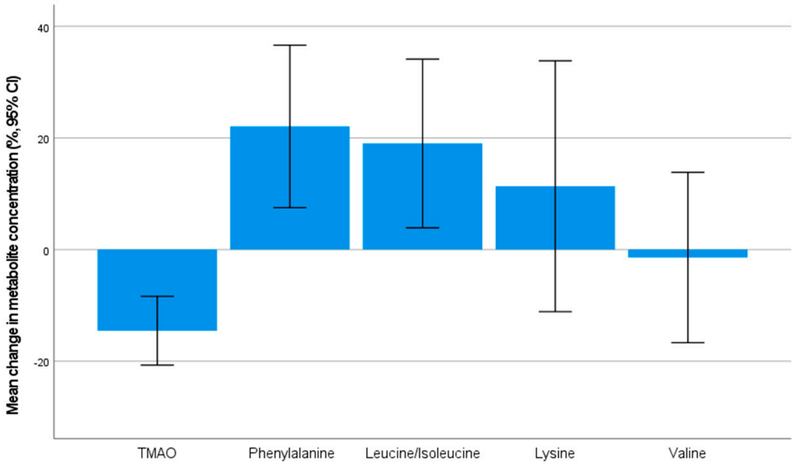

Figure S8. 24-hour monitoring experiment, mean changes in metabolite concentrations between T_0 and T_1. Values are presented as mean (% with 95% CI). TMAO indicates trimethylamine N-oxide.

Table S1. Final added concentrations for samples used during validation (Val) as well as quality control (QC).

Sample	Betaine [µmol/l]	Carnitine [µmol/l]	Choline [µmol/l]	TMAO [µmol/l]
Val 1	42.7	31.0	9.60	1.33
Val 2	85.4	62.0	28.8	6.66
QC 1	85.4	62.0	48.0	6.66

TMAO indicates trimethylamine *N*-oxide.

Table S2. Mass transitions (m/z) of all analytes and internal standards. The source temperature was set to 500 °C with curtain gas at 45 psi, nebulizer gas at 50 psi, and auxiliary gas at 70 psi. The ionspray voltage was set to 4000 V.

Analyte	Precursor m/z	Quantifier m/z	Qualifier m/z
TMAO	76	58	59
TMAO-d9	85	66	
Choline	104	60	58
Choline-d9	113	69	
Betaine	118	58	59
Betaine-d9	127	68	
Carnitine	162	103	60
Carnitine-d3	165	103	

TMAO indicates trimethylamine *N*-oxide.

Table S3. 24-hour monitoring experiment, baseline characteristics.

**Characteristic**	**N=12**
Men, %	33.3
Age, yrs.	34 (26-46)
BMI, kg/m²	22.7 (21.6-29.4)
Current smoker, %	8.3
Diabetes mellitus, %	0
Hypertension, %	8.3
Cardiocascular diseases, %	0
Nutritional behavior, %	
Balanced	66.7
Mainly vegetarian	33.3
Cholesterol, mmol/L	4.07 (3.94-4.55)
Triglyceride, mmol/L	0.75 (0.65-1.13)
CRP, mg/L	3.14 (1.70-3.62)
White blood cell count, x10⁹/L	5.0 (4.3-5.7)
ALAT, µkat/L	0.36 (0.29-0.43)
ASAT, µkat/L	0.33 (0.27-0.40)
Creatinine, µmol/L	69.50 (62.50-82.75)
Glucose, mmol/L	5.27 (4.97-5.79)

Values are presented as median (interquartile range) unless otherwise stated. Samples were collected at the trial start (T_0). ALAT indicates alanine transaminase; ASAT, aspartate transaminase; BMI, body mass index; CRP, C-reactive protein.

Table S4. 24-hour monitoring experiment, TMAO and its precursors at each sampling point.

	T_0 (N=12)	T_1 (N=12)	T_2 (N=12)	T_3 (N=12)	T_4 (N=12)	T_5 (N=2)	T_6 (N=2)	T_7 (N=2)	T_8 (N=2)	T_9 (N=12)
TMAO, µmol/L	2.7 (2.0-4.4)	2.5 (1.7-3.4)	2.2 (1.5-2.9)	2.1 (1.4-2.5)	1.9 (1.5-2.2)	1.5	1.4	1.4	1.7	1.9 (1.5-2.8)
Betaine, µmol/L	31.2 (22.5-38.5)	30.6 (24.0-40.5)	31.9 (24.6-44.0)	29.0 (23.0-40.4)	29.6 (24.3-40.1)	33.1	32.7	37.6	36.6	27.0 (22.3-33.4)
Carnitin, µmol/L	36.8 (31.0-44.1)	40.9 (34.7-47.5)	43.0 (36.3-52.0)	39.6 (33.6-45.7)	41.5 (34.0-46.0)	42.0	38.0	44.3	41.0	37.1 (33.4-47.2)
Choline, µmol/L	5.3 (4.3-5.9)	5.6 (4.8-5.9)	5.7 (5.0-6.8)	5.1 (4.4-6.3)	5.4 (4.1-6.1)	3.4	3.5	4.1	4.2	5.4 (4.5-6.4)

Values are presented as median (interquartile range). TMAO indicates trimethylamine N-oxide.

Table S5. Platelet activation experiment (experimental series 1), changes in TMAO, precursors, and eicosanoids plasma concentrations after platelet activation with ristocetin according to Born.

	**Untreated sample (N=9)**	**Control sample (N=9)**	**Activated sample (N=9)**	**p-value**
Betaine, µmol/L	37.6 (34.2-42.3)	35.9 (33.0-41.0)	37.0 (33.2-42.7)	0.123 [A]<br>0.767 [B]<br>0.110 [C]
Carnitine, µmol/L	43.4 (41.6-44.3)	40.3 (40.3-42.1)	41.7 (40.4-44.5)	0.050 [A]<br>0.213 [B]<br>0.139 [C]
Choline, µmol/L	5.3 (5.2-5.9)	5.3 (4.6-6.0)	6.0 (5.3-6.4)	0.192 [A]<br>0.021 [B]<br>0.110 [C]
TMAO, µmol/L	1.2 (0.9-1.5)	1.2 (0.9-1.5)	1.2 (1.0-1.4)	0.406 [A]<br>0.859 [B]<br>0.441 [C]
TXB_2, pg/mL	230 (158-862)	368 (349-621)	32042 (25968-45568)	0.310 [A]<br>0.018 [B]<br>0.008 [C]
12-HHT, pg/mL	593 (305-2087)	1166 (977-1915)	109309 (83583-135027)	0.110 [A]<br>0.008 [B]<br>0.008 [C]
18-HETE, pg/mL	153 (135-190)	153 (130-193)	165 (154-186)	0.678 [A]<br>0.374 [B]<br>0.515 [C]
16-HETE, pg/mL	243 (206-296)	233 (204-327)	251 (212-313)	0.594 [A]<br>0.441 [B]<br>0.859 [C]
15-HETE, pg/mL	287 (249-337)	303 (268-399)	1561 (1466-2633)	0.028 [A]<br>0.008 [B]<br>0.008 [C]
12-HETE, pg/mL	3266 (1734-10428)	4056 (1606-7211)	82427 (60056-140851)	0.953 [A]<br>0.008 [B]<br>0.008 [C]
Tetranor-12-HETE, pg/mL	92 (84-98)	90 (80-97)	3038 (2242-3661)	0.678 [A]<br>0.008 [B]<br>0.008 [C]
12-oxo-ETE, pg/mL	-	-	501 (464-629)	-<br>-<br>-
11-HETE, pg/mL	169 (146-197)	189 (166-227)	2354 (1777-3893)	0.086 [A]<br>0.008 [B]<br>0.008 [C]

8-HETE, pg/mL	209 (176-256)	205 (183-272)	486 (405-688)	0.110 A<br>0.008 B<br>0.008 C
5-HETE, pg/mL	368 (322-448)	467 (446-705)	731 (584-766)	0.015 A<br>0.008 B<br>0.028 C
13-HODE, pg/mL	6165 (5053-9696)	5838 (4987-9816)	6998 (5569-10519)	0.594 A<br>0.008 B<br>0.011 C
9-HODE, pg/mL	3653 (1886-5598)	3600 (1807-5560)	4178 (2214-7188)	0.678 A<br>0.008 B<br>0.008 C
12-HEPE, pg/mL	175 (108-307)	170 (115-233)	1139 (957-1811)	0.463 A<br>0.018 B<br>0.018 C
14,15-DHET, pg/mL	358 (337-439)	374 (332-431)	369 (337-451)	0.594 A<br>0.110 B<br>0.594 C
11,12-DHET, pg/mL	337 (334-374)	337 (322-370)	355 (328-371)	0.374 A<br>0.859 B<br>0.110 C
8,9-DHET, pg/mL	185 (132-197)	157 (134-236)	189 (175-235)	0.441 A<br>0.038 B<br>0.173 C
5,6-DHET, pg/mL	125 (106-143)	135 (116-158)	152 (132-182)	0.260 A<br>0.011 B<br>0.011 C
LA, ng/mL, pg/mL	12183 (6578-16258)	10167 (6747-18701)	13632 (7232-21023)	0.012 A<br>0.008 B<br>0.012 C
a/g LA, ng/mL	1135 (674-1417)	1069 (705-1509)	1259 (724-1498)	0.086 A<br>0.038 B<br>0.086 C
DHGLA, ng/mL	396 (290-1239)	340 (289-1206)	486 (329-986)	0.889 A<br>0.594 B<br>0.779 C
ARA, ng/mL	1094 (686-1472)	1081 (782-1698)	1522 (955-1778)	0.012 A<br>0.008 B<br>0.012 C
EPA, ng/mL	339 (322-368)	344 (325-396)	365 (361-388)	0.484 A<br>0.110 B<br>0.161 C
DHA, ng/mL	727 (545-1013)	760 (564-1081)	786 (586-987)	0.374 A<br>0.515 B<br>0.953 C

Values are presented as median (interquartile range). P values of continuous variables were calculated using the Wilcoxon test.

[A] Control samples were compared with untreated samples.

[B] Activated samples were compared with untreated samples.

[C] Activated were compared with control samples.

TMAO indicates trimethylamine-N-oxide; TXB_2, thromboxane B2; HHT, hydroxy-heptadecatrienoic acid; HETE, hydroxy-eicosatetraenoic acid; ETE, eicosatetraenoic acid; HODE, hydroxy-octadecadienoic acid; HEPE, hydroxy-eicosapentaenoic acid; DHET, dihydroxy-eicosatrienoic acid; LA, linoleic acid; DHGLA, dihomo-γ-linolenic acid; ARA, arachidonic acid; EPA, eicosapentaenoic acid; DHA, docosahexaenoic acid; PG, prostaglandin.

Table S6. Platelet activation experiment (experimental series 2), changes in TMAO, precursors, and eicosanoids plasma concentrations after platelet activation with ristocetin according to Born.

	**Untreated sample (N=7)**	**Control sample (N=7)**	**Activated sample (N=7)**	**p-value**
Betaine, µmol/L	32.9 (19.6-33.3)	31.6 (18.8-33.4)	30.4 (19.0-33.2)	0.233 [A]<br>0.499 [B]<br>0.866 [C]
Carnitine, µmol/L	42.8 (30.4-52.1)	42.8 (29.2-48.5)	44.8 (28.9-47.6)	0.249 [A]<br>0.866 [B]<br>0.866 [C]
Choline, µmol/L	6.2 (4.7-6.9)	6.4 (4.9-7.1)	7.0 (5.9-7.2)	0.866 [A]<br>0.176 [B]<br>0.176 [C]
TMAO, µmol/L	2.2 (1.9-7.9)	2.3 (1.9-7.4)	2.2 (1.8-7.3)	0.397 [A]<br>0.398 [B]<br>0.735 [C]
TXB_2, pg/mL	259 (229-483)	485 (417-715)	41951 (31615-115270)	0.173 [A]<br>0.028 [B]<br>0.028 [C]
12-HHT, pg/mL	706 (594-1026)	1209 (1024-1638)	138011 (73113-263846)	0.091 [A]<br>0.018 [B]<br>0.018 [C]
18-HETE, pg/mL	155 (143-251)	150 (131-249)	183 (145-281)	0.128 [A]<br>0.398 [B]<br>0.237 [C]
16-HETE, pg/mL	252 (226-308)	256 (214-363)	280 (255-344)	0.398 [A]<br>0.310 [B]<br>0.398 [C]
15-HETE, pg/mL	237 (233-358)	276 (243-342)	2187 (1851-5056)	0.063 [A]<br>0.018 [B]<br>0.018 [C]

12-HETE, pg/mL	4144 (3407-11111)	4479 (3366-6706)	148284 (77055-226390)	0.398 A<br>0.018 B<br>0.018 C
Tetranor-12-HETE, pg/mL	77 (63-130)	70 (62-113)	3302 (2025-3508)	0.398 A<br>0.018 B<br>0.018 C
12-oxo-ETE, pg/mL	-	-	750 (549-834)	-<br>-<br>-
11-HETE, pg/mL	186 (143-295)	176 (164-272)	3625 (2521-8700)	0.176 A<br>0.018 B<br>0.018 C
8-HETE, pg/mL	216 (205-288)	215 (192-264)	589 (556-826)	0.499 A<br>0.018 B<br>0.018 C
5-HETE, pg/mL	434 (258-685)	463 (245-517)	804 (467-1037)	0.866 A<br>0.128 B<br>0.176 C
13-HODE, pg/mL	4723 (4060-5616)	4810 (4361-5819)	7180 (5345-8300)	0.237 A<br>0.028 B<br>0.028 C
9-HODE, pg/mL	2694 (2465-5732)	2997 (2618-5595)	5537 (3572-8742)	0.499 A<br>0.018 B<br>0.018 C
12-HEPE, pg/mL	159 (134-458)	203 (143-273)	2147 (1802-4459)	0.499 A<br>0.018 B<br>0.018 C
14,15-DHET, pg/mL	363 (262-556)	349 (258-543)	366 (269-570)	0.612 A<br>0.176 B<br>0.237 C
11,12-DHET, pg/mL	312 (258-477)	325 (253-470)	331 (255-487)	0.499 A<br>0.499 B<br>0.398 C
8,9-DHET, pg/mL	173 (149-225)	155 (153-216)	213 (141-254)	0.028 A<br>0.398 B<br>0.310 C
5,6-DHET, pg/mL	129 (113-150)	130 (110-185)	164 (139-200)	0.237 A<br>0.176 B<br>0.398 C
LA, ng/mL, pg/mL	14990 (14066-21348)	17648 (11559-21135)	18808 (13037-24114)	0.735 A<br>0.398 B<br>0.237 C
a/g LA, ng/mL	1063 (845-1401)	1212 (700-1389)	1253 (880-1569)	0.499 A<br>0.310 B<br>0.237 C

DHGLA, ng/mL	392 (345-430)	377 (319-425)	429 (372-475)	1.000 [A]<br>0.398 [B]<br>0.176 [C]
ARA, ng/mL	1193 (987-1391)	1164 (900-1409)	1462 (1307-2555)	0.866 [A]<br>0.176 [B]<br>0.176 [C]
EPA, ng/mL	342 (317-425)	299 (280-425)	375 (360-496)	0.063 [A]<br>0.176 [B]<br>0.176 [C]
DHA, ng/mL	950 (776-1696)	976 (701-1871)	1078 (934-2218)	0.398 [A]<br>0.310 [B]<br>0.237 [C]

Values are presented as median (interquartile range). P values of continuous variables were calculated using the Wilcoxon test.

[A] Control samples were compared with untreated samples.

[B] Activated samples were compared with untreated samples.

[C] Activated were compared with control samples.

TMAO indicates trimethylamine-N-oxide; TXB_2, thromboxane B2; HHT, hydroxy-heptadecatrienoic acid; HETE, hydroxy-eicosatetraenoic acid; ETE, eicosatetraenoic acid; HODE, hydroxy-octadecadienoic acid; HEPE, hydroxy-eicosapentaenoic acid; DHET, dihydroxy-eicosatrienoic acid; LA, linoleic acid; DHGLA, dihomo-γ-linolenic acid; ARA, arachidonic acid; EPA, eicosapentaenoic acid; DHA, docosahexaenoic acid; PG, prostaglandin.

Table S7. Platelet activation experiment (experimental series 3), changes in eicosanoids plasma concentrations after platelet activation with ristocetin or ADP according to Born.

Activator	Untreated samples	Control samples	Activated samples: ADP				Activated samples: Ristocetin			
**TMAO-spike, μmol/L**			0	5	20	100	0	5	20	100
TXB_2, pg/mL	33	866	25400	18400	19900	21900	30000	33900	32000	25400
12-HHT, pg/mL	210	2080	57800	41600	45100	55900	69900	77300	67000	50600
18-HETE, pg/mL	41	29	43	35	53	36	39	49	38	36
16-HETE, pg/mL	118	121	122	132	124	118	125	124	131	121
15-HETE, pg/mL	229	298	1390	1050	955	1160	1280	1510	1200	966
Tetranor-12(S)-HETE, pg/mL	81	95	337	326	352	330	1820	1820	2040	1770
12-oxo-ETE, pg/mL	-	115	294	226	218	177	293	391	318	306
11-HETE, pg/mL	92	175	1600	1210	1130	1490	1590	1830	1540	1060
8-HETE/12-HETE, pg/mL	325	2140	18100	15500	16900	16800	23300	30600	24500	20400
5-HETE, pg/mL	244	282	395	350	376	370	331	349	314	342
13-HODE, pg/mL	7380	7290	7840	7490	8150	8040	7360	7580	7600	7870

9-HODE, pg/mL	4490	4850	6430	6220	6080	5560	5870	6800	6400	6900
12-HEPE, pg/mL	20	92	312	239	240	255	434	584	521	367
14,15-DHET, pg/mL	345	385	380	381	386	372	368	386	378	395
11,12-DHET, pg/mL	368	383	363	378	376	372	346	372	381	384
8,9-DHET, pg/mL	122	126	119	115	120	114	128	120	127	114
5,6-DHET, pg/mL	52	54	57	62	70	55	53	58	59	61
LA, pg/mL	19827	22004	22059	23816	22933	24063	19435	23108	21134	21030
a/g-LA, ng/mL	789	803	839	866	873	870	762	808	779	797
DHGLA, ng/mL	32	30	60	101	62	66	32	99	76	103
ARA, ng/mL	616	763	957	988	890	1029	769	927	940	971
EPA, ng/mL	70	81	87	89	77	87	89	82	92	84
DHA, ng/mL	545	577	572	613	586	584	588	576	613	596

Values are presented as median. TMAO indicates trimethylamine-N-oxide; TXB_2, thromboxane B2; HHT, hydroxy-heptadecatrienoic acid; HETE, hydroxy-eicosatetraenoic acid; ETE, eicosatetraenoic acid; HODE, hydroxy-octadecadienoic acid; HEPE, hydroxy-eicosapentaenoic acid; DHET, dihydroxy-eicosatrienoic acid; LA, linoleic acid; DHGLA, dihomo-γ-linolenic acid; ARA, arachidonic acid; EPA, eicosapentaenoic acid; DHA, docosahexaenoic acid; PG, prostaglandin.
